# Restoration of Chemism

**Published:** 1886-01

**Authors:** 


RATIONALISM IN MEDICAL TREATMENT, OR THE RESTORATION OF CHEMISM.—THE SYSTEM OF THE FUTURE. *By* WILLIAM THORNTON. *Boston. 1885. pp. 46.*

What the writer of this essay calls a new system is described by him as, "The internal treatment of diseases by chemicals of like nature to those that are found within the body in a healthy state."

This is the nearest approach he makes to committing himself to anything in the disconnected mass of verbiage here presented. The title is misleading, since there is no system formulated in the pages of the book. It contains simply a loose string of meaningless expressions, which the writer has evidently been turning over, for some time, in what he is pleased to term his mind.

Whether he is simply a man of weak intellect, honestly believing in his own vaporings, or a shrewd quack, seeking to advertise himself by his oddity, is not apparent. The little volume, though handsomely printed, has an uncanny look, from the fact that the leaves are printed only on one side. E. W. A.

